# Use of flow diverter device in basilar artery for aneurysm treatment: Case series and literature review

**DOI:** 10.3389/fneur.2022.990308

**Published:** 2022-08-17

**Authors:** Chuanchuan Wang, Deyuan Zhu, Xiaolong Xu, Yu Zhou, Rui Zhao, Qiang Li, Pengfei Yang, Qinghai Huang, Yi Xu, Jianmin Liu, Yibin Fang

**Affiliations:** ^1^Neurovascular Center, Changhai Hospital, Naval Medical University, Shanghai, China; ^2^Department of Neurovascular Disease, School of Medicine, Shanghai Fourth People's Hospital, Tongji University, Shanghai, China

**Keywords:** flow diverter, intracranial aneurysm, basilar artery, endovascular treatment, literature review

## Abstract

**Background:**

Flow diverters (FDs) for the treatment of basilar artery (BA) aneurysms remain controversial. In this study, we report our initial experience of flow diversion for treatment of this pathology.

**Methods:**

Consecutive patients with an aneurysm of the BA that was treated by implantation of the FD were included in our retrospective study. Procedural complications, aneurysm occlusion, and a functional outcome were evaluated. FD placement in BA for aneurysm treatment reported in the literature was also reviewed and summarized.

**Results:**

Sixteen patients with 16 BA aneurysms were treated by FD implantation with (*n* = 8) or without (*n* = 8) adjunctive coiling. The Tubridge was used in 13 (81.3%) and Pipeline in 3 (18.8%) procedures. Average aneurysm size was 15.7 mm. Four aneurysms were located at the basilar apex, six at the basilar trunk, and six at the vertebrobasilar junction. Three patients experienced procedural complications (18.8%), including two ischemic strokes and one hydrocephalus, with resultant mortality in one case (6.3%). Median follow-up was 7.7 months and available for 15 aneurysms. Complete/near-complete occlusion was seen in 13 (86.7%) aneurysms.

**Conclusion:**

In our initial experience, flow diversion is feasible and safe in the treatment of BA aneurysms with promising occlusion rates at mid-term follow-up. Larger cohort studies are required to validate these results.

## Introduction

Posterior circulation aneurysms remain an ongoing challenge to treat by either endovascular or surgical strategies due to the complexity of these lesions (1). Overall, endovascular methods have yielded slightly better results than microsurgery and were considered to be the primary treatment modality (2). Recently, flow diverters (FDs) have become reliable tools used to treat complex aneurysms. Many of these devices appear in studies on off-label use in basilar artery (BA) for aneurysm treatment, but published series are still limited and report heterogeneous results (3–5). Among the most commonly used is the Pipeline FD (Medtronic-Covidien, USA), as well as the Flow-Redirection Endoluminal Device (FRED; Microvention-Terumo, USA), Surpass (Stryker Neurovascular, USA), Silk (Balt Extrusion, France), p64 (Phenox, Germany), while others, such as the Tubridge (MicroPortNeuroTech, China), have not yet appeared in studies with such locations (6–9).

To date, two kinds of FDs have been commercially available in China: Tubridge and Pipeline. The Tubridge FD has proved to be a safe and effective device in managing complex aneurysms of anterior circulation, and its use was approved by the Chinese Food and Drug Administration in 2018 (10). We have used Tubridge and Pipeline devices to treat BA aneurysms since 2018. Herein, we present a single-center case series of patients with aneurysms located in the BA that were treated by flow diversion using the Tubridge and Pipeline. In addition, we present a comprehensive literature review focused on the safety and efficacy of FD placement in BA for aneurysm treatment.

## Materials and methods

### Study design

We performed a retrospective review of all the patients who underwent FD implantation in BA for aneurysm treatment between April 2018 and April 2021. This study was conducted with the approval of our Institutional Review Board. Given that patient data were collected in a deidentified manner and posed no risk to the patients, individual informed consent was not required or sought. Indications for using FDs include aneurysms with a high risk of failure with conventional endovascular or surgical methods, with recurrences, and fusiform, dissecting and large aneurysms with mass effect. Dolichoectatic aneurysms were not included in this treatment cohort. Patient demographic data, clinical presentation, aneurysm morphology, therapeutic strategy, complication, immediate angiographic and clinical result, and clinical and radiological follow-up information were determined from the electronic medical records.

### Procedure details

The patients were started on dual antiplatelet therapy with aspirin 100 mg daily and clopidogrel 75 mg daily at least 3 days before their scheduled procedure. Platelet function testing was performed routinely using thrombelastograms (TEG Hemostasis System, Haemoscope Corporation, USA) before the procedure. Ticagrelor (a loading dose of 180 mg followed by 90 mg two times daily) was substituted for the patients non-responsive to clopidogrel. After the procedure, dual antiplatelet therapy was continued at least 3 months, followed by a lifelong use of aspirin 100 mg daily. Additional periprocedural Tirofiban was used at the discretion of the operators based on procedural findings.

The Tubridge and Pipeline procedures were performed in the manner previously described (10–12). Transfemoral access was routinely used in all cases. Access through both femoral arteries was used when a “jailed” catheter was required for adjunctive coil placement or the plan was to sacrifice one of the vertebral arteries (VAs) in cases in which there was flow from the other VA directly into the aneurysmal sac. The decision of whether to use coils in combination with FD was considered in specific scenarios (i.e., for very large aneurysms, if the FD was prone to herniate into the aneurysm without coiling protection, or when persistent inflow jet impingement to the aneurysm dome existed before treatment). Overlapping FD devices implantation was considered in case that inflow jet into the aneurysm remained after single-device deployment or the single device was not sufficient to cover the entire lesion segment of dissecting and fusiform aneurysms. For FDs with poor adherence, a micro-guidewire was used in combination with a microcatheter to “massage” the FD device, or a balloon was used to expand the FD device. The type of FDs used during the procedure was determined by the operators.

### Procedural assessment and follow-up

Procedure-related complications, including hemorrhagic and thromboembolic events, were recorded. Symptomatic complications were defined as those associated with transient or permanent neurological deficits. A clinical outcome was evaluated at the baseline (presentation), discharge, and during follow-up according to the modified Rankin Scale (mRS) score.

Aneurysm occlusion was graded as completely occluded (100%), near-completely occluded with neck remnant (>90%), or incompletely occluded (<90%). Follow-up was performed regularly by digital subtraction angiography (DSA) at 3–12 months after treatment. If the patient declined DSA, cross-sectional imaging (CT angiography or MR angiography) was performed instead. Patency of parent arteries and jailed branches were also evaluated on follow-up angiogram.

### Literature review

The literature was reviewed based on a PubMed search of all cases with the use of FDs in the BA for aneurysm treatment, including the following keywords, singly and in combination: flow diverter, basilar artery, posterior circulation, aneurysm. We excluded literature with a small volume of cases and case reports. Data from large series (≥10 cases with FD placement in BA) published were selected and summarized.

### Statistical analyses

Statistical analyses were performed using SPSS 19.0 (SPSS, Chicago, IL, USA). Normally distributed continuous variables are presented as means and ranges. Categorical variables are presented as numbers with frequency.

## Results

### Baseline patient and aneurysm characteristics

A total of 16 BA aneurysms were treated with FD devices in 16 patients. The individual overview about the baseline, aneurysm, and procedural characteristics is presented in Table 1. The average age was 47 years old, and majority (62.5%) of the patients were men. Common presenting symptoms included headache or dizziness (50%), retreatment for recurrence (18.8%), ischemic stroke (12.5%), mass effect (6.3%), while 12.5% were incidental. Three recurrent cases had previously undergone endovascular treatment, including conventional stent assisted with coiling (*n* = 2) and PED implantation with coiling (*n* = 1) at outside hospitals. Of the 16 patients, 14 were mRS 0, one was mRS 1, and one was mRS 3 at presentation. Aneurysm morphology was classified as dissecting (50%), fusiform (31.3%), or saccular (18.8%), and the median aneurysm diameter was 15.7 mm, with 25% of aneurysms >20 mm. Four aneurysms were located at the basilar apex, six at the basilar trunk, and six at the vertebrobasilar junction. Platelet function testing was performed in 14 (87.5%) patients, and three clopidogrel non-responders were found and were substituted with ticagrelor.

**Table 1:** Patient demographics, aneurysm characteristics, and treatment details.

**Pt #**	**Age, y (Sex)**	**Presentation**	**mRS**		**Aneurysm characteristics**		**Treatment details**				**Angiographic FU**				
				**Previous Treatment**	**Location**	**Diameter, mm**	**Number and Type of FDs used**	**Additional Devices applied**	**Jailed Major Branches**	**Compli** **cations**	**Time**	**Moda** **lity**	**Occlu** **sion Grade**	**Jailed Branches**	**mRS at FU**
1	46 (M)	Dizziness	0		VBJ	20.2	2 TB	Coils	2 AICA		12	DSA	CO	Patent	0
2	56 (M)	Dizziness	0		VBJ	14.5	1 TB	Coils			6	DSA	NC		0
3	67 (M)	Incidental	0		Trunk	10.2	1 TB		1 AICA	Ischemic stroke	9	MRA	CO	Patent	1
4	66 (M)	Ischemic stroke	1		VBJ	20.4	1 TB	Coils		Mortality					6
5	57 (M)	Finding tandem BA aneurysms after infarction	0	LEO	Trunk	11.8	1 TB	Post-dilation with Scepter balloon	2 AICA		5	DSA	CO	Patent	0
6	8 (F)	Recurrence after previous treatment	0	LEO + coils	Tip	27.4	2 TB	Pre-dilation with Gateway balloon, Coils	1 PCA and 2 SCA		12	DSA	CO	All occluded	0
7	28 (F)	Recurrence after previous treatment	0	PED + coils	VBJ	17.5	1 TB	Coils			7	DSA	NC		0
8	68 (F)	Dizziness	0		Trunk	11.1	1 TB		1 AICA		7	DSA	IC	Patent	0
9	52 (F)	Dizziness	0		Tip	9.1	1 TB		2 SCA		7	DSA	CO	Patent	0
10	56 (M)	Dizziness	0		Trunk	13.6	1 TB		1 AICA		10	DSA	CO	Patent	0
11	38 (F)	Headache	0		Trunk	5.8	1 TB		1 PCA and 2 SCA		7	DSA	NC	Patent	0
12	65 (M)	Headache	0		VBJ	18.1	1 TB	Coils	2 AICA		9	DSA	NC	Patent	0
13	27 (F)	Incidental	0		Trunk	10.5	1 TB	Coils	1 PCA and 2 SCA		7	DSA	CO	Patent	0
14	25 (M)	Headache	0		VBJ	9.7	1 PED				6	CTA	CO		0
15	77 (M)	Progressivebrainstem compression syndrome	3		Tip	32.6	1 PED	Coils	1 PCA and 2 SCA	Progressive hydrocephalus	3	DSA	IC	Patent	4
16	36 (M)	Recurrence after previous treatment	0	LVIS + coils	Tip	18.3	1 PED		1 PCA and 2 SCA		9	DSA	CO	1 PCA occluded	0

BA, basilar artery; CO, complete occlusion; EP, enterprise stent; FD, flow diverter; FU, follow-up; IC, incomplete occlusion; LV, LVIS stent; NC, near-complete occlusion; PCA, posterior cerebral artery; PED, pipeline; Pt, patient; SCA, superior cerebellar artery; TB, Tubridge; UE, upper extremity; VBJ, vertebrobasilar junction. #, number.

### Procedural details and angiographic outcome

**Figure 1:**
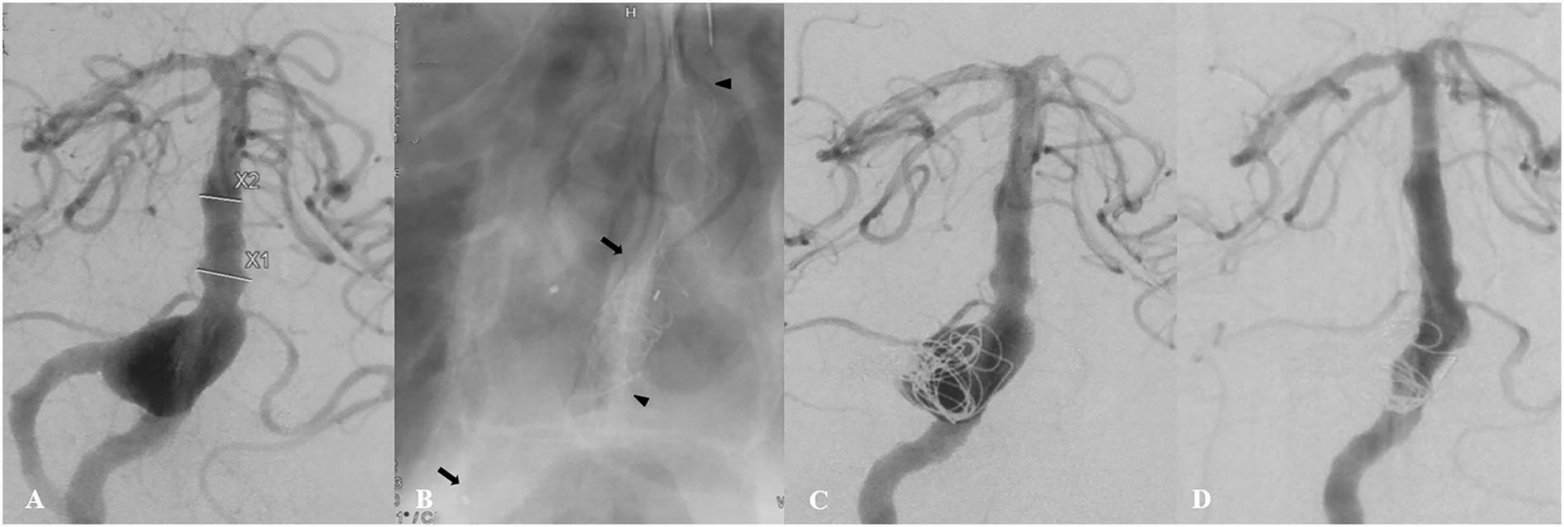
A 46-year-old patient (Case 1) presented with a 2-month history of dizziness. Preoperative angiography showed a large fusiform vertebrobasilar junction aneurysm **(A)**. This patient underwent placement of two telescoping Tubridge devices (the arrow and the arrowhead), along with coiling and right vertebral artery sacrifice **(B,C)**. Follow-up angiography 12 months later showed complete aneurysm occlusion **(D)**.

Procedural success was achieved in all cases. In eight patients (50%), aneurysms were additionally coiled. Most (87.5%) aneurysms were treated by single FD placement using 11 Tubridge and three Pipeline devices, while the remaining two cases were treated with two Tubridge devices, one of which was treated with an overlapping technique and the other telescoping (Figure 1). No obvious difficulties with device delivery or deployment were encountered. Balloon angioplasty was performed before or after FD deployment in two cases, respectively. There were 26 covered branches after implantation of the FD, including nine anterior inferior cerebellar arteries (AICAs), twelve superior cerebellar arteries (SCAs), and five posterior cerebral arteries (PCAs). At the end of the procedure, no acute occlusion of covered branches was found.

**Figure 2:**
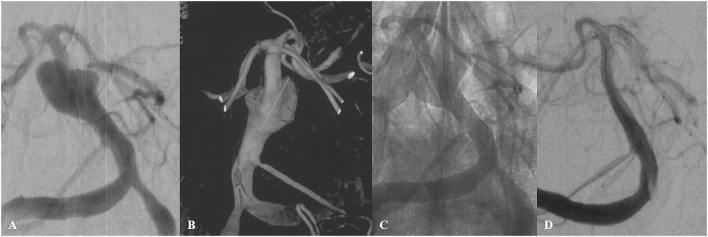
A 56-year-old male patient (Case 10) with a large basilar trunk aneurysm presented with dizziness. Preoperative angiography **(A)** with 3-dimensional reconstruction **(B)** demonstrated an irregular dissecting aneurysm at the middle basilar trunk. A single Tubridge device was placed in the basilar trunk **(C)**. 10-month follow-up angiography demonstrated complete obliteration of the aneurysm **(D)**.

During a mean follow-up of 7.7 months (range 3–12 months), angiographic follow-up was available for 15 of 16 aneurysms at different time intervals. Thirteen patients were followed-up with DSA, one with MRA, and one with CTA. Complete or near-complete aneurysm occlusion was observed in nine (60%) and four (26.7%) aneurysms, respectively. The remaining two aneurysms with incomplete occlusion revealed progressive occlusion at follow-up. Aneurysm recanalization was not observed. At the follow-up, of all 26 vessels covered by the device, 22 were patent, while other four branches were occluded asymptomatically, including two PCAs and two SCAs. Figure 2 shows a representative case illustration of a patient successfully treated with a Tubridge device for a large basilar trunk aneurysm.

### Procedural complications and clinical outcome

Procedural complications developed in three patients (18.8%), including two ischemic strokes and one progressive hydrocephalus, resulting in one mild neurologic deficit, one severe neurologic deficit, and one death (6.3% mortality). There were zero intracranial hemorrhages or SAH. No inhospital mortality occurred. One patient was found dead at 12-month telephone follow-up (Case 4). This patient initially presented with vertigo and limb numbness. MR imaging found infarctions at thalamus and cerebellum. DSA demonstrated a 20-mm fusiform vertebrobasilar aneurysm involving bilateral vertebral artery. This patient was noted to have extensive atherosclerosis and aneurysmal ectasia. Uneventful single Tubridge placement with adjuvant coiling of the aneurysm lumen was performed, and the left vertebral artery was also sacrificed. After that, the patient missed MRI/angiography follow-up due to non-compliance. This patient was also non-compliant with antiplatelet drugs. 5 months after treatment, the patient suffered a loss of consciousness and coma. Emergent CT showed a massive brainstem infarction, but no further details were available. We speculate that stent occlusion secondary to antiplatelet non-compliance may be a contributing factor. This patient died ultimately in a local hospital.

One delayed thromboembolic event developed in an additional patient (Case 3) who experienced hemiplegia and hypoesthesia 3 months after treatment of a 10-mm basilar trunk aneurysm using single Tubridge deployment. Emergent DSA demonstrated a patent basilar artery and visible perforators. MRI disclosed pontine infarction and the patient was discharged home with an mRS score of 1.

A 77-year-old male patient (Case 15) presented with dizziness and unsteady gait on admission and was found with a giant basilar tip aneurysm. The patient gradually became unconscious after implantation of a single Pipeline device and adjunctive coils. The CT scan conformed a third ventricular mass leading obstructive hydrocephalus. A ventriculoperitoneal shunt was inserted for the patient with an unfavorable outcome at discharge (mRS = 4).

Clinical follow-up at 8–18 months (mean, 12.2 months) was achieved in 15 patients because one patient died during follow-up. One patient suffering obstructive hydrocephalus after treatment was still disabled with an mRS score of 4 at 8-month clinical FU. Except this case, a favorable clinical outcome (mRS ≤ 2) was observed in other 14 patients (87.5%).

### Literature review

A literature review regarding the use of FDs in BA identified 296 cases within 11 original case reports/case series (Table 2). The overall complication rate was 27%, mainly ischemic events (15.8%). The mortality rate was 11.8%. Among 164 cases with angiographic follow-up, 123 (75%) cases achieved complete or near-complete occlusion.

**Table 2:** A summary of large series (≥10 patients) with flow diverter implantation in the basilar artery for aneurysm treatment.

**Author, published year**	**FD types**	**All BA cases**	**Complications (%)**	**Ischemic (%)**	**Hemorrhagic (%)**	**Mass effect (%)**	**Others (%)**	**Mortality (%)**	**CO/NC at FU** **(total FU cases, %)**
Ge et al. (13)	PED	29	13 (44.8)	7 (24.1)	3 (10.3)	3 (10.3)	0 (0)	7 (24.1)	20 (23, 87)
Hellstern et al. (9)	p64	30	6 (20)	4 (13.3)	2 (6.7)	0 (0)	0 (0)	1 (3.3)	20 (26, 76.9)
Wallace et al. (15)	PED	15	2 (13.3)	1 (6.7)	0 (0)	0 (0)	1 (6.7)	1 (6.7)	10 (13, 76.9)
Bender et al. (16)	PED	25	4 (16)	3 (12)	0 (0)	1 (4)	0 (0)	2 (8)	NA
Dmytriw et al. (18)	PED/FRED	16	3 (18.8)	0 (0)	2 (12.5)	1 (6.3)	0 (0)	1 (6.3)	11 (16, 68.8)
Griessenauer et al. (19)	PED	68	21 (30.9)	NA	NA	NA	NA	8 (11.8)	50 (68,73.5)
Taschner et al. (7)	Surpass	26	NA	NA	NA	NA	NA	8 (30.8)	NA
Lopes et al. (20)	PED	44	9 (20.4)	6 (13.6)	1 (2.3)	1 (2.3)	1 (2.3)	5 (11.4)	NA
Wakhloo et al. (21)	Surpass	10	4 (40)	2 (20)	0 (0)	0 (0)	2 (20)	2 (20)	5 (6, 83.3)
Phillips et al. (22)	PED	21	6 (28.6)	4 (19.0)	2 (9.5)	0 (0)	0 (0)	0 (0)	NA
Kulcsár et al. (23)	SILK	12	5 (41.7)	5 (41.7)	0 (0)	0 (0)	0 (0)	0 (0)	7 (12, 58.3)
Sum		296							
Number (sum, %)			73 (270, 27)	32 (202, 15.8)	10 (202, 5)	6 (202, 3)	4 (202, 2)	35 (296, 11.8)	123 (164, 75)

FD, flow diverter; CO, complete occlusion; FU, follow-up; NA, data not available; NC, near-complete occlusion.

## Discussion

In this study, we examined the feasibility, safety, and efficacy profile of the Tubridge and Pipeline FD in the treatment of BA aneurysms. The FD device was successfully implanted in the BA for all 16 aneurysms. Procedural complications occurred in 18.8% of the patients. Complete or near-complete occlusion was achieved in 86.7% of aneurysms at follow-up of 7.7 months. There were no instances of aneurysm recurrence or retreatment. Our initial experience showed satisfactory results with acceptable clinical outcomes and high occlusion rates after mid-term follow-up.

### The tubridge flow diverter device

The Tubridge FD is a braided, self-expanding device with flared ends, which has various features that seem to predetermine its use in the posterior circulation. The application of a nickel–titanium alloy allows for improved shape-holding memory and super-elasticity. The platinum–iridium material used for the radiopaque microfilaments improves visualization of the stent during deployment. More importantly, the large-size Tubridge (> 3.5 mm), which was mostly used in the posterior circulation, has more braided microfilaments, which decreases the shortening rate after its full opening and offers more appropriate pore attenuation (24).

Previously, a multicenter, prospective, randomized, controlled clinical trial (PARAT study) has verified the safety and efficacy of the Tubridge in unruptured large and giant intracranial aneurysms (10). In the Tubridge group with 82 enrolled subjects, the complete occlusion rates were 75.34% at 6-month imaging follow-up. Device-related morbidity and mortality occurred in 12.19 and 4.88% of the patients. Only 4 subjects with posterior circulation aneurysms were included in this study and were not discussed in detail. Subsequently, the application of Tubridge has been reported in other different subtypes, such as recurrent aneurysms, middle cerebral artery aneurysms, and cavernous carotid artery aneurysms (17, 25, 26). The safety and the efficacy of using Tubridge in BA aneurysms have not been evaluated.

In the current study, the Tubridege FD was mainly used, and the Pipeline was only used in three cases. Given the limitations of sample size, we were unable to make any direct comparison between the two groups. Nevertheless, our data showed the safety and the efficacy of the Tubridge in treatment of BA aneurysms. Symptomatic thromboembolic complications occurred in 15.5% (2/13) of the patients, resulting in one death, with a mortality rate of 7.7%. Complete or near-complete occlusion was achieved in 91.7% (11/12) of aneurysms at a median follow-up of 24 months. A favorable functional outcome was achieved in all cases.

### Safety of flow diverters in basilar artery

Aneurysms in the basilar artery are characterized by complex neurovascular anatomy with life-sustaining perforating vessels arising from the lesion and the adjacent vessel along the brainstem. Placement of FDs inevitably results in coverage of these side branches, which further exposes the patient to thromboembolic and ischemic complications (27). As shown in our literature review, available studies reporting on the use of FDs in the BA show highly variable results, with complication rates ranging from 13.3 to 44.8%. One of the important observations from the overall outcomes is that the mean procedural complication rate appears to be still high (27%), which leads to the mean mortality rate of 11.8%. Thromboembolic events are the main source of poor outcomes. Ischemic stroke is mainly associated with invisible perforator infarction, jailed vessel occlusion, and stent occlusion. The occlusion of invisible perforator may be the most common cause of ischemia (16). In the present case series, the rates of procedural complications and mortality were 18.8 and 6.3%, respectively. Ischemic stroke was the most common complication, with an incidence rate of 12.5%, including perforator infarction and stent occlusion one patient each. Although two PCAs and two SCAs were invisible at the follow-up angiogram, these jailed branches were occluded asymptomatically.

Several factors associated with complications and outcomes have been identified. Firstly, FD may be more appropriate for asymptomatic patients or patients with mild symptoms due to the poorer outcomes related to the treatment of aneurysms in patients with higher baseline mRS scores (7). Secondly, implantation of multiple FD devices was prudent in the series, which helped to reduce thromboembolic complications (20). Longer devices with larger diameters were necessary for spanning of the fusiform segment, reliable opening, and improved apposition. Furthermore, rigorous platelet function testing and subsequent regimen adjustments were critical factors to mitigate neurologic complications from FD procedures (6, 14). In our study, platelet function was assessed in 87.5% of procedures, and the rate of antiplatelet regimen adjustment was 21.4%. Applying those principles in the current study may have explained the very favorable safety profile.

Hemorrhagic events are relatively uncommon. In the literature review, the pooled hemorrhagic complication rate among the 202 patients was 5%. In this study, no hemorrhagic event was occurred. Adjunctive coiling may provide protection from bleeding complications by altering intra-aneurysmal hemodynamics and controlling thrombosis (28). One patient who experienced progressive hydrocephalus postoperatively had symptoms of brainstem compression before FD treatment. Worsening of mass effect was less common in our pooled analysis, which demonstrated a rate of 3%. Several studies have shown that rapid thrombus formation after FD treatment and subsequent thrombus renewal, instead of thrombus organization, may induce an autolytic and inflammatory cascade, causing edema and further weakening the arterial wall, leading to IA expansion and aggravation of mass effect (29, 30).

### Efficacy of posterior circulation aneurysms with flow diverters

The literature review looking at treatment of BA aneurysms with FDs reported complete/near-complete occlusion rates, ranging from 58.3 to 87% (mean, 75%). These findings are consistent with our results with an occlusion rate of 86.7%, following treatment with Tubridge and Pipeline devices. Still, our data are promising in terms of occlusion of some of the most challenging cerebral aneurysms. Also, follow-up was limited to 7.7 months on average; long-term follow-up would show higher occlusion.

Previous reports have summarized a variety of predictors of occlusion. A predictor of occlusion in BA aneurysms was age, with older aneurysms occluding less often (19). The use of adjunctive coils has been associated with increased occlusion rates (31). Aneurysms harboring large amounts of pre-treatment thrombus were associated with lower rates of complete occlusion (32). Large or giant aneurysm size correlated with aneurysm persistence for posterior circulation aneurysms (16, 19).

Our study has various inherent limitations. A major limitation is the retrospective design. There may have been a selection bias during patient sampling. More specifically, BA aneurysms are admittedly heterogeneous, while, in this study, several types were absent, such as acutely ruptured aneurysms, perforator aneurysms, and dolichoectatic aneurysms. The literature review was relatively simple; the relatively small sample size also precludes statistical analysis; therefore, an independent meta-analysis and larger-scale studies are needed. However, the results of our preliminary experience of the treatment of BA aneurysms using the Tubridge and Pipeline devices are encouraging.

## Conclusion

Although no definitive conclusions can be drawn from this case series because of the small number of treated patients, the Tubridge and Pipeline FD seem to be safe and effective for the treatment of BA aneurysms at mid-term follow-up. However, studies with a long-term follow-up and larger series are necessary to validate these results.

## Data availability statement

The original contributions presented in the study are included in the article/supplementary material, further inquiries can be directed to the corresponding authors.

## Ethics statement

Written informed consent was obtained from the minor(s)' legal guardian/next of kin for the publication of any potentially identifiable images or data included in this article.

## Author contributions

CW, DZ, and XX: analysis and interpretation of data. YZ, RZ, QL, PY, QH, and YX: review and editing. YF and JL: overall review and study design. CW: the draft of the work. All authors contributed to the article and approved the submitted version.

## Funding

This work was supported by the National Natural Science Foundation of China (82071279 and 82001262), the Shanghai Pujiang Program (2019PJD051), the Shanghai Sailing Program (20YF1448300), and the Key Scientific Research Project supported by Changhai Hospital, Naval Medical University (2020YSL004).

## Conflict of interest

The authors declare that the research was conducted in the absence of any commercial or financial relationships that could be construed as a potential conflict of interest.

## Publisher's note

All claims expressed in this article are solely those of the authors and do not necessarily represent those of their affiliated organizations, or those of the publisher, the editors and the reviewers. Any product that may be evaluated in this article, or claim that may be made by its manufacturer, is not guaranteed or endorsed by the publisher.
